# Nonvolatile and reconfigurable two-terminal electro-optic duplex memristor based on III-nitride semiconductors

**DOI:** 10.1038/s41377-024-01422-4

**Published:** 2024-03-29

**Authors:** Zhiwei Xie, Ke Jiang, Shanli Zhang, Jianwei Ben, Mingrui Liu, Shunpeng Lv, Yang Chen, Yuping Jia, Xiaojuan Sun, Dabing Li

**Affiliations:** 1 State Key Laboratory of Luminescence and Applications, Changchun Institute of Optics, Fine Mechanics and Physics, Chinese Academy of Sciences, Dongnanhu Road No. 3888, Changchun, 130033 China; 2 Center of Materials Science and Optoelectronics Engineering, University of Chinese Academy of Sciences, Yuquan Road No. 19, 100049 Beijing, China

**Keywords:** Optical data storage, Optical sensors

## Abstract

With the fast development of artificial intelligence (AI), Internet of things (IOT), etc, there is an urgent need for the technology that can efficiently recognize, store and process a staggering amount of information. The AlScN material has unique advantages including immense remnant polarization, superior temperature stability and good lattice-match to other III-nitrides, making it easy to integrate with the existing advanced III-nitrides material and device technologies. However, due to the large band-gap, strong coercive field, and low photo-generated carrier generation and separation efficiency, it is difficult for AlScN itself to accumulate enough photo-generated carriers at the surface/interface to induce polarization inversion, limiting its application in in-memory sensing and computing. In this work, an electro-optic duplex memristor on a GaN/AlScN hetero-structure based Schottky diode has been realized. This two-terminal memristor shows good electrical and opto-electrical nonvolatility and reconfigurability. For both electrical and opto-electrical modes, the current on/off ratio can reach the magnitude of 10^4^, and the resistance states can be effectively reset, written and long-termly stored. Based on this device, the “IMP” truth table and the logic “False” can be successfully reproduced, indicating the huge potential of the device in the field of in-memory sensing and computing.

## Introduction

With the fast development of artificial intelligence (AI), Internet of things (IOT), etc, there is an urgent need for the technology that can efficiently recognize, store and process a staggering amount of information^1–6^. Traditional data processing based on Von Neumann architecture generally separates the sensing, storage and computing units, and thus massive data will be transported among the units, causing the bottleneck of storage wall^7,8^. The in-memory sensing and computing devices, wherein an optical signal can be detected and stored for calculation and the calculated results can be re-stored in the same unit, are promising to break the bottleneck^9^. The memristors can realize the in-memory computing and numerous materials can be applied to prepare memristors including two-dimensional materials like transition metal sulfides, inorganic phase change materials like oxides and perovskites, and ferroelectric materials like In_2_Se_3_^10–20^. Moreover, some of the memristors are photosensitive, which can be implemented to in-memory sensing and computing^14,17,18,21^.

Although the above materials provide terrific opportunities for realizing in-memory sensing and computing, the material scale, stability, and quality cannot be effectively guaranteed at present from the real application perspective. To choose a photosensitive and storable material that is compatible with the current mainstream semiconductor materials and processes is of great significance for the concept implementation of in-memory sensing and computing. As a ferroelectric material, AlScN has unique advantages including immense remnant polarization and superior thermal stability^22,23^, which is a good storage material. Moreover, the AlScN is very easy to integrate with III-nitrides, which are very mature in terms of both material and device fabrication process, due to their similar element composition and crystal structure. Recently, great progress has been made for AlScN materials, such as the ultrathin AlScN memristor, and the scalable CMOS back-end-of-line-compatible AlScN-based ferroelectric transistor by wafer level fabrication process^24–30^.

However, because of the large band-gap, strong coercive field, and low photo-generated carrier generation and separation efficiency^23,31^, it is difficult for the AlScN itself to accumulate enough photo-generated carriers at the surface/interface to induce polarization inversion^18,32,33^. Hence, it is necessary to construct a photosensitive AlScN memristor for in-memory sensing and computing. As a representative of the third generation semiconductors, GaN presents excellent photoelectric characteristics such as direct band-gap, strong ultraviolet absorption, and high carrier mobility, and is widely applied to detection, light-emission and optoelectronic integration^34–38^. Besides, as mentioned above, AlScN is easy to integrate with GaN to form hetero-structure due to their similar element composition and crystal structure. Moreover, GaN can form strong polarization hetero-structure with AlScN because it possesses strong spontaneous and piezoelectric polarization, providing great space for built-in field regulation. Thus, to combine the ferroelectric AlScN with photosensitive GaN to form hetero-structure is promising to realize high performance memristor, taking advantages of the high industrial maturity of III-nitrides to promote the in-memory sensing and computing.

Here, we fabricate a two-terminal nonvolatile and reconfigurable III-nitride based electro-optic duplex memristor. The device is an n-GaN/i-GaN/AlScN hetero-structure based Schottky diode, in which the i-GaN and AlScN determine the photosensitive and storage characteristics, respectively. For both electrical and opto-electrical modes, the current on/off ratio can reach the order of 10^4^, and the resistance states can be effectively reset, written and long-termly stored. At electrical mode, the resistance state change originates from the ferroelectric polarization inversion of the AlScN layer in the hetero-structure, which influences the depletion region width and electron transport barrier height of the device. At opto-electrical mode, the memory window can be controlled by the illuminating light intensity due to the photoconductive effect of the i-GaN layer and the photoinduced electron transport barrier reduction effect, which can regulate the partial bias applied to the AlScN layer. Based on this device, the material implication (IMP) truth table and the logic “False” can be successfully reproduced, indicating the huge potential of our device in the field of in-memory sensing and computing.

## Results

**Fig. 1:**
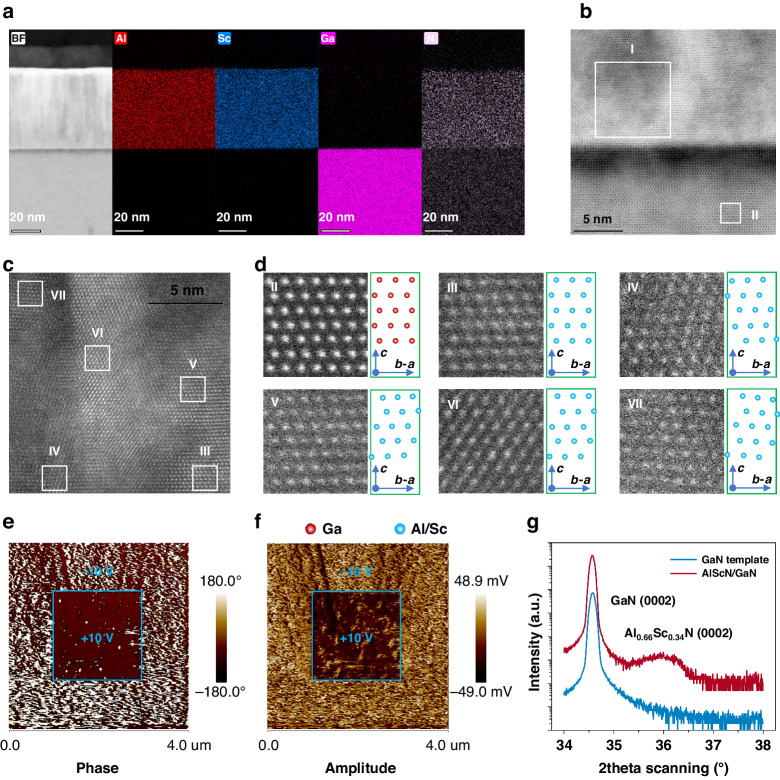
Characterization of the GaN/AlScN hetero-structure. **a** BF-STEM image and corresponding Al, Sc, Ga, N element EDS mappings of the i-GaN/AlScN hetero-structure. **b** HR-BF-STEM image near the GaN/AlScN interface. **c** HR-HADDF-STEM image of the area marked by the white box I in (**b**). **d** Enlarged HR-HADDF-STEM images of the areas marked by the white box II–VII in (**b**, **c**) and their corresponding atomic arrangement. All STEM images are captured along <11-20> direction. **e**, **f** Scanning PFM phase and amplitude images of the epilayer after applying a bias of +10 V and −10 V, respectively. **g** XRD 2theta scanning for the GaN/AlScN epilayer and the reference GaN template

To fully utilize the photosensitive property of the GaN material and the ferroelectric property of the AlScN material, a hetero-structure composed of undoped GaN/AlScN (i-GaN/AlScN) is designed. The i-GaN layer thickness is ~300 nm and the AlScN layer thickness is ~60 nm. The hetero-structure is grown on a thick n-type GaN/sapphire template by metal-organic chemical vapor deposition (MOCVD) and magnetron sputtering, respectively, to form the n-GaN/i-GaN/AlScN heterojunction (see “Materials and methods” section). Figure 1a exhibits the bright field (BF) scanning transmission electron microscope (STEM) image along <11-20> direction and its corresponding Al, Sc, Ga, N element X-ray energy dispersive spectroscopy (EDS) mappings of the i-GaN/AlScN hetero-structure. It can be clearly seen that the hetero-structure indeed contains GaN and AlScN layers and the AlScN layer thickness is actually ~60 nm^39^.

To investigate the crystal structure of the hetero-structure, high-resolution (HR) BF-STEM image along <11-20> direction near the interface is captured as shown in Fig. 1b, from which we can observe that the i-GaN layer has a relatively regular atomic arrangement, while the upper AlScN layer has a relatively chaotic atomic arrangement. To further confirm the atomic arrangement of the AlScN layer, a HR high-angle annular dark-field (HAADF) STEM image along <11-20> direction, as shown in Fig. 1c, is taken in the area marked by the while box I in Fig. 1b. It can be seen that there are obvious grain boundaries. Figure 1d shows the enlarged HR-HAADF-STEM images of the areas marked by the while boxes II–VII in Fig. 1b, c and their corresponding atomic arrangement, successively. The i-GaN layer is strictly ***c***-oriented (Fig. 1d II), while different areas of the AlScN layer show different lattice orientations. The area III (Fig. 1d III) is ***c***-oriented, the area IV is ***c*** deviating ***a*-*b*** oriented (Fig. 1d IV), and the areas V ~ VII are ***c*** deviating ***b*-*a*** oriented with different deviation angles (Fig. 1d V–VII), respectively. These results demonstrate that the i-GaN layer has strict ***c***-oriented single crystal structure, while the AlScN layer has polycrystalline structure with ***c***-preferred orientation, which is mainly determined by the growth methods.

To verify the ferroelectricity of the prepared AlScN layer, the piezoelectric force microscope (PFM) domain writing test is conducted on the n-GaN/i-GaN/AlScN heterojunction. The scanning PFM phase and amplitude images after applying a bias of +10 V and −10 V are shown as Fig. 1e, f, respectively. Clear boundaries between different applying bias areas are observed in the images, demonstrating that different applying bias corresponds different ferroelectric polarization directions, and further indicating that the polarization direction of the AlScN layer can be changed by the applied bias within ±10 V, implying the potential to manufacture a memory device of the grown hetero-structure. The X-ray diffraction (XRD) 2theta scanning is employed to estimate the Sc content. As shown in Fig. 1g, the diffraction curve of the GaN/AlScN epi-wafer exhibits obvious peaks near 34.5° and 36.0°, which reflect the GaN (0002) plane and the AlScN (0002) plane, respectively^23^. Accordingly, the ***c*** lattice constant of the AlScN layer can be roughly calculated to be 4.9854 Å, from which the Sc content can be estimated to be about 34%^40,41^. In addition, the very wide full width at half maximum (FWHM) of the 2theta scanning curve further indicates the ***c***-preferred polycrystalline structure of the AlScN epilayer, as discussed above.

**Fig. 2:**
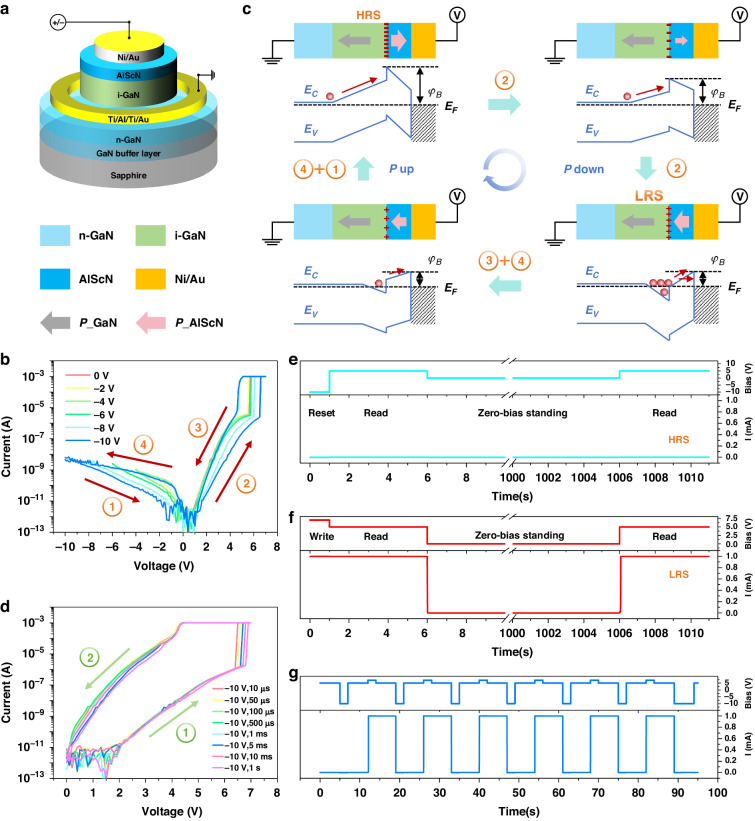
Electric driving ferroelectric polarization inversion characteristics of the GaN/AlScN Schottky diode. **a** Schematic diagram of the device. **b** IV hysteresis loops with initial scanning voltage from 0 to −10 V. **c** Schematic diagram of the variation of the AlScN ferroelectric polarization and device energy band with the applied bias. **d** IV hysteresis loops after electric pulse of −10 V from 10 μs to 1 s. **e**, **f** Retention properties of the device with rectangular electric reset and write pulses of –10 V and 7 V and read bias of 5 V. Pulse widths for reset, write and read are 1 s, 1 s, and 5 s, respectively. Storage time is 1000 s without bias. **g** Cyclically program and erase for the device with rectangular write and reset pulses of 7 V and −10 V and read bias of 5 V. Pulse widths for reset, write and read are 2 s, 2 s, and 5 s, respectively

Standard III-nitride based device fabrication process is employed to make the memristor (see “Materials and Methods” section). The schematic diagram of the device is shown in Fig. 2a. In reality, the device is a typical GaN/AlScN hetero-structure based Schottky diode. Figure 2b shows the current-voltage (IV) hysteresis loops of the device with different initial applied voltage from 0 V to −10 V, from which the memristor characteristics is displayed. Anticlockwise IV hysteresis is observed, which is due to the ferroelectric polarization inversion of the AlScN layer^42^. The higher the initial reverse bias, the higher the current explosion voltage and thus the wider the memory window, and even at an reverse bias of 10 V, the current explosion voltage is still not saturated. This result indicates that applying reverse bias to the device can induce the ferroelectric polarization inversion of the AlScN layer while an reverse bias of 10 V can not fully polarize the AlScN layer. Nonetheless, the bias of −10 V can still reset the device to high resistance state (HRS).

Figure 2c displays the schematic diagrams of the energy band structure and the polarization of the device at different stage in the IV hysteresis loop. In the initial case, corresponding to the process ① in Fig. 2b, reverse bias upward polarizes the AlScN layer, and negative polarization charges at the i-GaN/AsScN interface are induced, significantly increasing the electron potential barrier height φ_B_. The high barrier will impede the electron transport through the depletion region, and place the device in the HRS. With the increase of forward bias, corresponding to the process ② in Fig. 2b, the depletion region width shortens and the bias applied on the AlScN layer increases, resulting in weaker polarization in the AlScN layer and thus lower the φ_B_. The current changes from drift to diffusion and gradually increases. When the bias further increases, the electric field in the AlScN layer exceeds the coercive field, causing ferroelectric polarization inversion. The polarization direction of the AlScN layer changes downward and the interface polarization charges change from negative to positive, thus reducing the φ_B_. Due to the reduction of the depletion region width and the φ_B_, the device inverts to low resistance state (LRS) and the current increases abruptly. As the bias reduces, corresponding to the process ③ and part of process ④ in Fig. 2b, since the ferroelectric polarization direction of the AlScN layer is not changed, the device keeps LRS. At 5 V bias, the HRS to LRS ratio (on/off ratio) can reach ~10^4^. Further applying reverse bias, corresponding to part of the process ④ in Fig. 2b, the ferroelectric polarization in the AlScN layer undergoes inversion and the depletion region width and the φ_B_ again increase to the initial state. Eventually, the device returns to HRS and waits to enter the next cycle.

The IV hysteresis curves imply that the magnitude of the applied bias is crucial for the degree of the ferroelectric domain inversion in the AlScN layer, and the duration of the applied bias is crucial, too. Pulse bias of -10 V with different pulse widths is applied to initiate the device and the IV hysteresis curve under forward bias is measured to observe the memory window change, as shown in Fig. 2d. Before measurements, a bias pulse with magnitude of 7 V and width of 5 s is applied to polarize the AlScN layer to the same state. As is seen, with the increase of the pulse width from 10 μs to 1 s, the memory window gradually widens and the current explosion voltage increases from 6.4 V to 6.8 V. It proves that when the applied electric field exceeds the coercive field, the ferroelectric domain walls begin to move, but it will take a certain period to move sufficiently and then trigger polarization inversion. Nonetheless, a short pulse of 10 μs is sufficient to trigger partial polarization inversion of the AlScN layer and then to form wide enough memory window, indicating the huge application potential of the GaN/AlScN hetero-structure based Schottky diode type memristor.

Furthermore, to verify the storage performance of the device, retention properties at HRS and LRS are measured as shown in Fig. 2e, f, respectively. The reset and write electric rectangular pulses are set to be –10 V and 7 V and the read voltage is set to be 5 V, respectively, according to the IV hysteresis curves. Then the resistance state is stored for 1000 s at zero-bias. As is seen, the HRS and LRS can be effectively reset and write to the device, and resistance state can be successfully read after 1000 s zero-bias standing, demonstrating the good electrical nonvolatility. Figure 2g shows the cyclic programming and erasing process of the device, which exhibits good cycling characteristics and stability, demonstrating the good electrical reconfigurability.

**Fig. 3:**
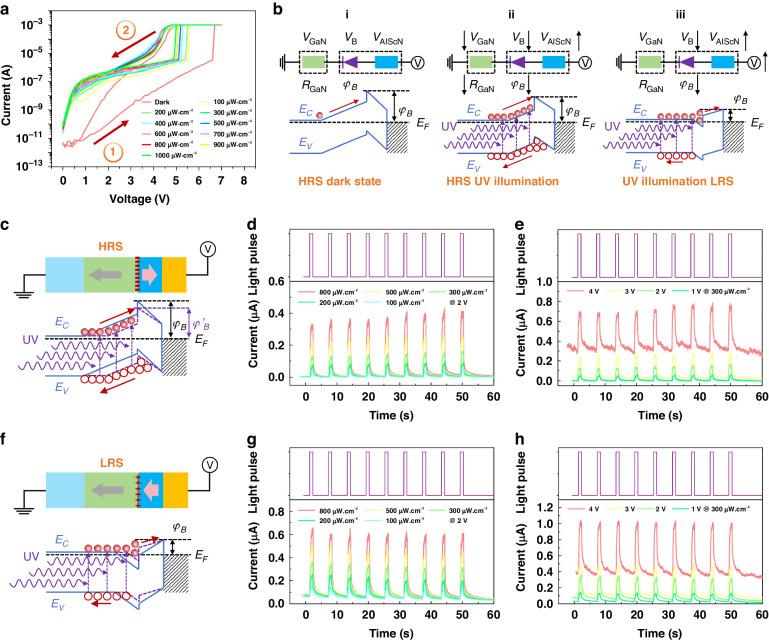
Photoinduced polarization inversion characteristics of the GaN/AlScN Schottky diode. **a** IV hysteresis loops measured with different light intensity from 100 to 1000 μW cm^−2^. A 355 nm-LED is used as the light source. Before measurements, the device is reset to HRS by electric rectangular pulse of −10 V and 5 s. **b** Schematic diagrams of the variation of the AlScN ferroelectric polarization and device energy band under illumination. **c**, **f** Schematic diagrams of the device energy band at HRS and LRS under illumination, respectively. **d**, **g** IT curves at HRS and LRS under different pulse light intensity at 2 V working bias. **e**, **h** IT curves at HRS and LRS under different working bias at 300 μW cm^−2^ UV light illumination

Since the GaN/AlScN hetero-structure based Schottky diode type memristor contains the GaN photosensitive material, it is very potential to employ light to control the memory characteristics for the device, namely realizing optically controlled memristor^43–46^. Therefore, the optical characteristics is measured by employing an ultraviolet (UV) light illumination using a light-emitting diode (LED) with a peak wavelength of 355 nm. The IV hysteresis curves in dark and under illumination with light intensity from 100 to 1000 μW cm^−2^ are shown in Fig. 3a. To ensure the consistency of the initial state of the device for each test, a rectangular pulse bias of −10 V and 5 s is applied to the device to polarize the AlScN layer. It is found that, compared to the dark state, the low-bias current under illumination is effectively increased and the photocurrent increases with the light intensity increasing. More importantly, compared to the dark state, the current explosion voltage is obviously reduced and continuously decreasing with the light intensity increasing. When the light intensity increases from 0 to 1000 μW cm^−2^, the current explosion voltage decreases from ~6.6 V to ~5 V. These results indicate that this device is optically controllable, as expected.

Figure 3b schematically depicts the optical control mechanism of the device. If the device is working at a relatively low bias that is not high enough to trigger the AlScN ferroelectric polarization inversion (i), for example 4 V, even the UV illumination can produce electron-hole pairs, increase the i-GaN conductance, reduce the depletion region width, and reduce the φ_B_, the photoinduced voltage increase upon the AlScN layer is still not high enough to trigger the inversion. Without the inversion, the device keeps HRS and the photoinduced current change is relatively small and cannot be stored (ii). If the device is working at a relatively high bias that is still not high enough to trigger the AlScN ferroelectric polarization inversion (i), for example 6 V, the photoinduced voltage increase upon the AlScN layer becomes high enough to trigger the inversion, inverting the device to LRS (iii). It is worth emphasizing that, even if the device works at a very low bias under which employing UV illumination cannot trigger the AlScN ferroelectric polarization inversion, the resistance state of the device is also related to the light intensity, due to the photoconductive effect and the φ_B_ variation.

At the HRS, as shown in Fig. 3c, the photo-generated carriers can effectively increase the i-GaN conductance, reduce the depletion region width, and reduce the φ_B_, thus altering the current. If the applied bias and the illuminated light intensity cannot induce the AlScN ferroelectric polarization inversion, once the illumination is removed, the device will recover to its original HRS. After setting the device to HRS by applying an electric rectangular pulse of −10 V and 5 s, the time-dependent current (IT) curves under 2 V bias at an UV light pulse width of 1 s with different intensity, and the IT curves at an UV light pulse with width of 1 s and intensity of 300 μW cm^−2^ under different bias, are measured as shown in Fig. 3d, e, respectively. At the LRS, as shown in Fig. 3f, the photo-generated carriers will mainly influence the i-GaN conductance and the barrier width, thus altering the current. Similarly, if the applied bias and the illuminated light intensity cannot induce the AlScN ferroelectric polarization inversion, once the illumination is removed, the device will recover to its original LRS. After setting the device to LRS by applying an electric rectangular pulse of 7 V and 5 s, the IT curves under 2 V bias at an UV light pulse width of 1 s with different intensity, and the IT curves at an UV light pulse with width of 1 s and intensity of 300 μW cm^−2^ under different bias, are measured as shown in Fig. 3g, h, respectively. For both HRS and LRS, it is found that when the light is off, the current rapidly decrease, demonstrating the photoinduced resistance state change cannot be long-termly stored without the AlScN ferroelectric polarization inversion.

**Fig. 4:**
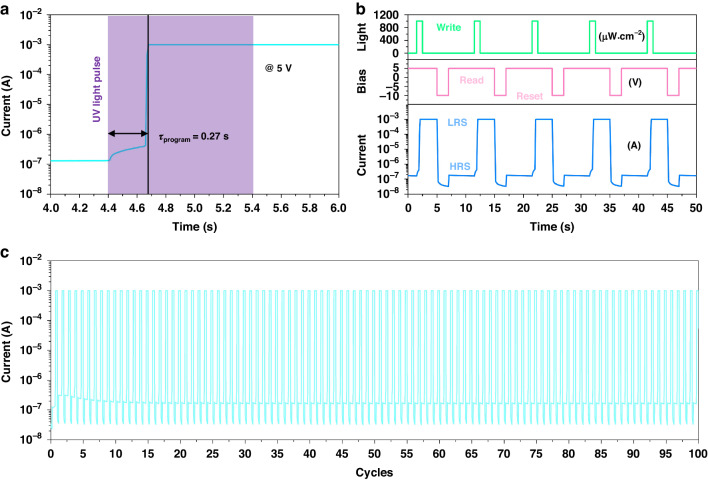
Optical writing and electrical erasing. **a** Time-resolved photoinduced polarization inversion process of the device at continuous voltage of 5 V and light pulse with width of 1 s and intensity of 1000 μW cm^−2^. **b** Opto-electrical programming process of the device. **c** Opto-electrical programming of the device for 100 cycles. During the programming, the write pulse light intensity and width are 1000 μW cm^−2^ and 1 s, and the read and reset voltages are 5 V and −10 V, respectively

According to the analyses above, it is confirmed that at working bias below 4 V, the polarization of the AlScN layer in the device will not reverse even the illumination light intensity reaches 1000 μW cm^−2^. Although the UV light illumination can momentarily change the device resistance state, the resistance state cannot be long-termly stored. Based on the dark and light IV hysteresis curves, an applying bias of 5 V with an illumination intensity of 1000 μW cm^−2^ may induce the polarization inversion. Hence, we first preset the device to HRS and then apply 5 V continuous bias to the device, during which an UV light pulse with intensity of 1000 μW cm^−2^ and width of 1 s is illuminated on the device. The IT curve is shown in Fig. 4a. It is found that without illumination, the output current keeps low level, and once the device is illuminated, the output current first rises slowly and then suddenly increases near four orders of magnitude to 10^−3^ A. It takes about 0.27 s for the device to complete this change. Since the AlScN ferroelectric polarization can be partially reversed by a short electric rectangular pulse of 10 μs and −10 V, it can be speculated that the time for the AlScN ferroelectric polarization inversion only occupy a small portion of the total time for the resistance change of the device. Combining the IT curve, it can be deduced that most of the time is devoted to regulate the bias applied on the AlScN layer through the photoconductive effect and the φ_B_ variation. It is worth noting that the pulse light is provided by a LED driven by a pulse power supply, so the response speed of the LED and power supply may also affect the IT curve. When the light turns off, the output current still keeps 10^−3^ A, namely the device successfully stores the LRS.

Figure 4b shows the opto-electrical programming process of the device. During the process, the write pulse light intensity and width are 1000 μW cm^−2^ and 1 s, and the read and reset voltages are 5 V and −10 V, respectively. As is seen, the device can be effectively written and reset by the light pulse and the electric pulse, respectively, and the programming process is repeatable. Within 100 times of the write-read-reset-read process, the device exhibiting excellent repeatability, as shown in Fig. 4c, demonstrating the good opto-electrical nonvolatility and reconfigurability.

Table 1 compares the configuration, material, structure, memory mechanism and on/off ratio of some reported memristors in recent years. Currently, the memristors that can simultaneously be electrically and optically controlled are based on 2D or oxide semiconductors. Our device, based on III-nitrides, can realize the electro-optic duplex working mode and achieve an on/off ratio of 10^4^ magnitude, which is relatively advanced. Moreover, different from the reported optically controlled ferroelectric memristors that require the interaction between the ferroelectric material itself and light to induce the polarization inversion, our device relies on the interaction between the other part of the structure and light to induce the ferroelectric part to generate polarization inversion, which provides a new direction for device structure design.

**Table 1:** Comparison between our device and some reported electrical or optical memristors

Configuration	Material/Structure	Memory mechanism	Switch	*λ* (nm)	On/off	Refs.
Three-terminal	MoS_2_/PZT	Ferroelectric polarization inversion	Electrical	/	10^4^	^10^
	In_2_Se_3_/BN/Gr	Photoinduced depolarization electric field shielding	Electrical optical	Visible light	10^4^	^33^
Two-terminal	MoO_x_	Ionic valence state change	Electrical optical	365	40	^9^
	O_D_-IGZO/O_R_-IGZO	Ionic valence state change	Optical	420/800	4	^14^
	α-In_2_Se_3_	Photoinduced depolarization electric field shielding	Electrical optical	900	10^4^	^18^
	n-GaN/AlScN	Ferroelectric polarization inversion	Electrical	/	10^4^–10^5^	^28^
	n-GaN/AlScN	Ferroelectric polarization inversion	Electrical	/	10^4^	^31^
	n-GaN/i-GaN/AlScN	Photoinduced bias change upon ferroelectric layer	Electrical optical	355	10^4^	This work

**Fig. 5:**
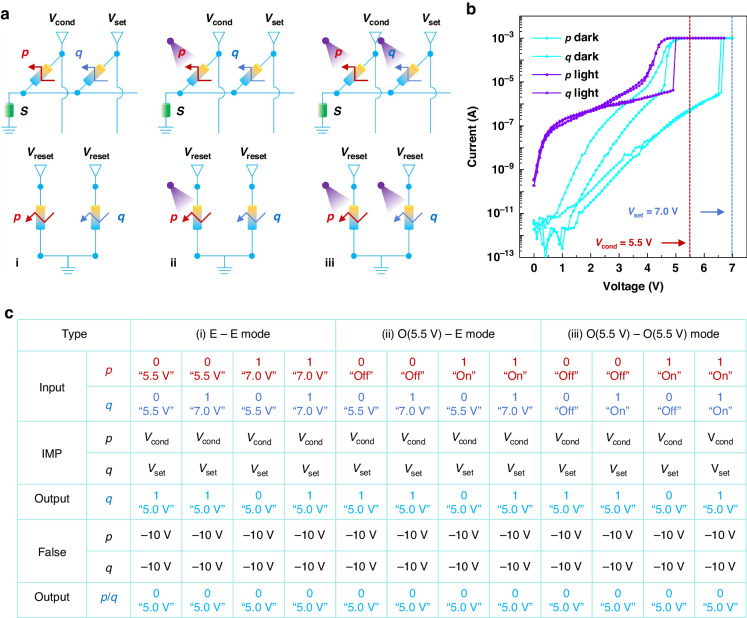
Logic operation strategy for the memristor. **a** Illustration of an in-memory IMP and False operation that can be written by E-E, O-E, and O-O modes, respectively. **b** IV hysteresis curves of the devices *p* and *q* in dark and on illumination under forward bias. **c** In-memory logic calculation and corresponding truth table under different working mode

Thanks to the excellent nonvolatility and reconfigurability of our device at both electrical and opto-electrical modes, in-memory sensing and computing can be operated. Figure 5a schematically displays the realization of the in-memory IMP and False operation by two devices *p* and *q* and an external resistance *s* at electrical-electrical (E-E) (i), optical-electrical (O-E) (ii), and optical-optical (O-O) (iii) modes, respectively. It is worth noting that the conductivity of the resistance *s* needs to be at least one order of magnitude higher than that of the devices *p* and *q* at HRS and one order of magnitude lower than that of the devices *p* and *q* at LRS. The result is stored in the device *q* for all operations^47,48^. Figure 5b shows the forward IV hysteresis curves of the devices *p* and *q* in the dark and on illumination. Before the tests, both devices are reset by an electric rectangular pulse of −10 V, 1 s and the illumination light intensity is 1000 μW cm^−2^. The devices show similar optical and electrical properties to the above analyzed devices. Accordingly, the IMP operation biases *V*_cond_ and *V*_set_ can be set as 5.5 V and 7.0 V, the False operation bias *V*_reset_ can be set as −10 V, and the read bias can be set as 5.0 V, respectively.

Here, we take the O-E working mode for example, in which the devices *p* and *q* are optically and electrically controlled, respectively. The HRS and LRS represent the logic “0” and “1”. In this mode, a bias of 5.5 V needs to be applied to the device *p*. Hence, for the device *p*, the “light-off” and “light-on” represent the input of logic “0” and “1”, and for the device *q*, the “5.5 V write bias” and “7.0 V write bias” represent the input of logic “0” and “1”, respectively. If the devices *p* and *q* are written “0” and “0”, the device *q* will change to LRS after the IMP operation, obtaining the logic “1”. If the devices *p* and *q* are written “0” and “1”, the device *q* will keep LRS after the IMP operation, obtaining the logic “1”. If the devices *p* and *q* are written “1” and “0”, the device *q* will keep HRS after the IMP operation based on Ohm’s law and Kirchhoff’s law, obtaining the logic “0”. If the devices *p* and *q* are written “1” and “1”, the device *q* will keep LRS after the IMP operation, obtaining the logic “1”. Meanwhile, for all input conditions, the device *q* will change to HRS after the False operation. Figure 5c summaries all the inputs and outputs after the IMP and False operation based on the device connection types in Fig. 5a for all three working modes. The corresponding key working parameters are also listed. As is seen, the “IMP” truth table and the logic “False” are successfully reproduced. We know that IMP logic and False logic can form a complete logical set and all 16 logic operations can be realized through cascading. Since the basic IMP and False operations can be realized, more complex logic calculations can be completed.

## Discussion

An electro-optic duplex memristor on a GaN/AlScN hetero-structure based Schottky diode has been realized. This two-terminal memristor shows good electrical and opto-electrical nonvolatility and reconfigurability. For both electrical and opto-electrical programming modes, the current on/off ratio can reach the magnitude of 10^4^, and the resistance states can be effectively reset, written and long-termly stored. At electrical mode, the resistance state change originates from the ferroelectric polarization inversion of the AlScN layer in the hetero-structure, which influences the depletion region width and electron transport barrier height of the device. At opto-electrical mode, the memory window can be controlled by the illuminating light intensity due to the photoconductive effect of the i-GaN layer and the photo-generated carrier induced electron transport barrier reduction effect, which can regulate the partial bias applied to the AlScN layer. Based on this device, the “IMP” truth table and the logic “False” can be successfully reproduced, indicating the huge potential of our device in the field of in-memory sensing and computing.

Due to the demand surge of the in-memory sensing and computing, various types of memristors and photomemristors have been developed successively. Nonetheless, the device here still owns advantages over the reported devices. First, the device can realize pure-electrical and opto-electrical duplex working modes, and under both modes the device exhibits good programmable memory properties. Then, the device has a two-terminal configuration, which is of benefit to some large-scale integration applications. Next, taking advantage of the wide application range and high industrial maturity of III-nitrides, the device can move toward mass production and application more quickly. Finally, the device relies on the interaction between one part of the structure and the illuminating light to induce the other ferroelectric part to generate polarization inversion, providing a new device design conception of combining the photosensitive materials to ferroelectric materials.

However, we realize there is still room for improvement in this work. First, due to the sputtering growth method of the AlScN layer, polycrystalline ferroelectric domains are formed, affecting the large-scale uniformity and reliability. Second, the device can only realize the “0/1” binary calculation and storage, limiting its application range. We are now trying to solve the two problems. We believe the epitaxial technology for AlScN, such as the molecular beam epitaxy, can solve the polycrystalline ferroelectric domains problem. As for the multiple-state storage, in addition to realizing single crystal ferroelectric layer, to construct AlScN multiple hetero-structures may also take effect.

## Materials and methods

### Materials growth

The structure is grown on ***c***-oriented sapphire substrate by MOCVD. During the growth, the TMGa and NH_3_ are used as the Ga and N precursors, the SiH_4_ is used as the n-doping sources, and the H_2_ is used as the carrier gas, respectively. First, the sapphire substrate is loaded to the growth chamber. Before the growth, the sapphire substrate is first thermally cleaned in a H_2_ and NH_3_ mixed gas atmosphere at 850 °C for 10 min to remove the surface contamination. Then, the temperature is increased to 900 °C to grow a GaN low-temperature nucleation layer of ~20 nm and further increased to 1080 °C to grow a GaN high-temperature buffer layer of ~1 μm. Next, an Si-doped n-type GaN contact layer of ~2 μm is grown to form an n-GaN/sapphire template. The electron concentration is ~2 × 10^19^ cm^−3^. After that, an unintentionally doped GaN layer (i-GaN) of ~300 nm is grown. Finally, the epi-wafer is unloaded from the MOCVD system and transferred to a RF-reactive magnetron sputtering system to grow an AlScN layer. A metal Al target and a metal Sc target are used as the Al and Sc sources, and the sputtering is in N_2_ atmosphere with the pressure of 0.7 Pa and temperature of 400 °C. The Al/Sc ratio is controlled by the target sputtering power. The AlScN layer is ~60 nm.

### Device fabrication

The devices are made by standard III-nitrides semiconductor device fabrication process. First, an SiO_2_ mask is grown on the epi-wafer by a plasma enhanced chemical vapor deposition (PECVD) system at 300 °C. Then, the device mesa patterns are transferred to the SiO_2_ mask by lithography and reactive ion etching (RIE), sequentially. Then, the wafer is etched by an inductive coupling plasma (ICP) system to expose the n-GaN layer and the residual SiO_2_ mask is removed by the 10% buffered oxide etch (BOE) solution. Next, the electrode on n-GaN of Ti-20 nm/Al-80 nm/Ti-50 nm/Au-70 nm is deposited by e-beam evaporation and thermal evaporation. The n-electrode is annealed at 600 °C for 30 s in N_2_ to form better ohmic contact. Finally, the electrode on AlScN of Ni-20 nm/ Au-80 nm is deposited by e-beam evaporation and thermal evaporation, forming Schottky contact.

### Characterizations and measurements

TEM-ready sample is prepared using the in situ focused ion beam (FIB) lift out technique on an FEI Dual Beam FIB/SEM. The sample is capped with sputtered C and e-Pt/I-Pt prior to milling. The TEM lamella is ~100 nm. The sample is imaged by an FEI Tecnai TF-20 FEG/TEM at 200 kV in bright-field (BF) STEM mode and HAADF-STEM mode. The STEM probe size is 1–2 nm in nominal diameter. EDS mappings are acquired on an Oxford INCA Bruker Quantax EDS system. The PFM images is measured by an Bruker Multimode 8 equipment. The PFM tip is applied with a bias of −10 V to scan a square region. At the center of the scanned region, a bias of +10 V is applied to scan a square region, causing different regions to undergo different polarization inversion process. A Bruck D8 discover HRXRD is employed to estimate the Al/Sc ratio and epilayer crystal quality. The IV and IT curves are recorded by a PDA FS-Pro 380 semiconductor analyzer. During the IV and IT measurements, the maximum current is limited to 10^−3^ A to protect the device. The UV light is provided by a commercial UV LED with a peak wavelength of 355 nm. The LED is driven by a DH1766 DC Power Supply.
